# The evolutionary advantage of fitness‐dependent recombination in diploids: A deterministic mutation–selection balance model

**DOI:** 10.1002/ece3.6040

**Published:** 2020-01-27

**Authors:** Sviatoslav Rybnikov, Zeev Frenkel, Abraham B. Korol

**Affiliations:** ^1^ Institute of Evolution University of Haifa Haifa Israel; ^2^ Department of Evolutionary and Environmental Biology University of Haifa Haifa Israel; ^3^ Department of Mathematics and Computational Science Ariel University Ariel Israel

**Keywords:** diploids, fitness dependence, purifying selection, recombination, recombination modifier

## Abstract

Recombination's omnipresence in nature is one of the most intriguing problems in evolutionary biology. The question of why recombination exhibits certain general features is no less interesting than that of *why it exists at all*. One such feature is recombination's fitness dependence (FD). The so far developed population genetics models have focused on the evolution of FD recombination mainly in haploids, although the empirical evidence for this phenomenon comes mostly from diploids. Using numerical analysis of modifier models for infinite panmictic populations, we show here that FD recombination can be evolutionarily advantageous in diploids subjected to purifying selection. We ascribe this advantage to the differential rate of disruption of lower‐ versus higher‐fitness genotypes, which can be manifested in selected systems with at least three loci. We also show that if the modifier is linked to such selected system, it can additionally benefit from modifying this linkage in a fitness‐dependent manner. The revealed evolutionary advantage of FD recombination appeared robust to crossover interference within the selected system, either positive or negative. Remarkably, FD recombination was often favored in situations where any constant nonzero recombination was evolutionarily disfavored, implying a relaxation of the rather strict constraints on major parameters (e.g., selection intensity and epistasis) required for the evolutionary advantage of nonzero recombination formulated by classical models.

## Graphical abstract

An amazing feature of recombination is fitness dependence (FD). The so far developed models have focused on the evolution of FD recombination mainly in haploids, although the empirical evidence for this phenomenon comes mostly from diploids. Using numerical analysis of modifier models for infinite panmictic populations, we show here that FD recombination can be evolutionarily advantageous in diploids subjected to purifying selection. We ascribe this advantage to the differential rate of disruption of lower‐ versus higher‐fitness genotypes, which can be manifested in selected systems with at least three loci.

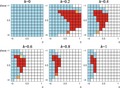

## 1 INTRODUCTION

Meiotic recombination is a process of reshuffling the parental genetic material, which takes place when a sexual organism produces its gametes. For about a century, recombination's omnipresence in nature has been a most intriguing question, given the evolutionary ambiguity of this process (Bell, 1982; Fisher, 1930; Maynard Smith, 1971; Weismann, 1889). Indeed, on the one hand, the new allelic combinations generated in this process serve as raw material to meet selection demands. On the other hand, it can also breakdown the existing combinations, including even the most successful ones. However, the question of why recombination exhibits certain general features is no less interesting than the initial question of why recombination exists at all (Korol, Preygel, & Preygel, 1994; Lenormand, Engelstädter, Johnston, Wijnker, & Hang, 2016). One such feature is recombination's sensitivity to external and/or internal conditions affecting the proportion of recombinants in the progeny. Harold Plough was the first to show that recombination rates (RRs) can rise when the organism is exposed to an ecological stressor (Plough, 1917). Empirical studies provide accumulating evidence for the ecological plasticity of recombination (for recent reviews, see Bomblies, Higgins, & Yant, 2015; Modliszewski & Copenhaver, 2017; Stapley, Feulner, Johnston, Santure, & Smadja, 2017). Earlier, it was noticed that recombination's ecological plasticity is genotype‐specific (Elliott, 1955; Nakamura, 1966; Wilson, 1959), although the pattern of such specificity remained obscure. In 1986, Zhuchenko, Korol, Gavrilenko, and Kibenko (1986) demonstrated that stressor‐induced changes in RRs can be modulated by genotype fitness in a negative‐feedback manner so that less stress‐tolerant genotypes show more pronounced increases in RR. Moreover, RR may be sensitive to fitness even when no ecological stressors are imposed, that is, when variation in fitness among individuals results from their differential genetic background (e.g., deleterious mutations) rather than from their differential stress tolerance (Tucić, Ayala, & Marinković, 1981). In general, one can think of “fitness‐dependent” (Zhuchenko et al., 1986) or “fitness‐associated” (Agrawal, Hadany, & Otto, 2005) recombination; herein, we use the former term, abbreviated as FD recombination. Empirical evidence for this phenomenon is still very limited, for both stressor‐induced (Aggarwal et al., 2019; Hunter, Huang, Mackay, & Singh, 2016; Jackson, Nielsen, & Singh, 2015; Khlebova, 2010; Kilias, Alahiotis, & Onoufriou, 1979; Korol et al., 1994; Zhong & Priest, 2011; Zhuchenko et al., 1986) and mutation‐induced (Tedman‐Aucoin & Agrawal, 2012; Tucić et al., 1981) changes in RR. Importantly, this evidence comes from diploids—mainly from fruit flies (Aggarwal et al., 2019; Hunter et al., 2016; Jackson et al., 2015; Kilias et al., 1979; Korol et al., 1994; Tedman‐Aucoin & Agrawal, 2012; Tucić et al., 1981; Zhong & Priest, 2011), but also from plants, such as tomato (Zhuchenko et al., 1986) and wheat (Khlebova, 2010).

An intriguing question is whether FD recombination can be considered as an evolvable phenotype. Analysis of natural populations infers that variation in RRs may indeed be adaptive (Ritz, Noor, & Singh, 2017). Theoretical models have clearly demonstrated the evolutionary advantage of FD recombination in haploids (Agrawal et al., 2005; Gessler & Xu, 2000; Hadany & Beker, 2003a, 2003b; Wexler & Rokhlenko, 2007). At that, the advantage arose in situations that seem to be qualitatively different (see Discussion), namely when genotype fitness affects (a) RR between the modifier locus and the selected system, and (b) RR within the selected system. Although diploid population genetics models usually behave similarly to haploid ones, extending the above‐mentioned results for FD recombination to diploids appeared to be nontrivial. Specifically, the evolutionary advantage of FD recombination in the first situation was shown to be impossible; while in the second situation, it required additional assumptions, such as different RRs in the coupling‐ versus repulsion‐phase double heterozygotes (Agrawal et al., 2005). In contrast, a recent study showed that FD recombination can be evolutionarily advantageous in diploids under cyclical selection, with genotype fitness affecting RRs within the selected system (Rybnikov, Frenkel, & Korol, 2017). Similar results were obtained in diploid models with cyclical selection where ecological stressors increased RR within the selected system (Rybnikov et al., 2017; Zhuchenko, Korol, Preigel, & Bronstein, 1985).

To address the discrepancy between the results obtained in the aforementioned diploid‐selection models, that is, between the “positive” result of Zhuchenko et al. (1985) and the “negative” result of Agrawal et al. (2005), it was suggested that more complex selection regimes, such as cyclical selection, favor FD recombination more than less complex ones, such as directional selection or mutation–selection balance (Agrawal et al., 2005). Alternatively, the evolutionary advantage/disadvantage of FD recombination in the considered models might be affected by the presence or absence of variation in fitness among genotypes (Rybnikov et al., 2017). Obviously, variation in fitness should concern only those genotypes in which recombination may affect the population structure in the next generation, that is, genotypes heterozygous for at least two selected loci; in all other genotypes, RRs are “immaterial,” in terms of Otto and Barton (1997) (we refer to genotypes heterozygous for at least two selected loci as “recombination‐responsive”). To further explore this assumption, here, we examine the evolution of FD recombination in diploids under mutation–selection balance, which is a relatively simple selection regime compared with the cyclical selection. The wild‐type genotype is assumed to have the highest fitness, while mutations at any locus are deleterious. In the deterministic infinite‐population models, if mutations at different loci affect fitness in a purely multiplicative way, recombination is known to be neutral. However, if the presence of mutant alleles simultaneously at several loci decreases fitness more radically (synergistic epistasis), then recombination can appear evolutionarily advantageous (Barton, 1995; Charlesworth, 1990; Feldman, Christiansen, & Brooks, 1980; Gabriel, Lynch, & Bürger, 1993; Kondrashov, 1984; Lynch, Conery, & Burger, 1995; Otto & Barton, 1997; Otto & Feldman, 1997).

Here, we test whether FD recombination can be favored over constant RR in a deterministic mutation–selection balance model. First, we test whether FD recombination can be favored in cases where constant recombination is disfavored (Section 3.2). Second, we test whether FD recombination can evolutionarily displace constant recombination in cases where the latter is favored (Section 3.3). In all these tests, we compare the examined recombination strategies using the modifier approach (Kimura, 1956; Nei, 1967), that is, based on the dynamics of selectively neutral recombination‐modifying alleles.

## 2 MODELS AND METHODS

### 2.1 Life cycle

We consider an infinite population of obligate sexual diploids with total panmixia. The life cycle includes random mating, selection at the diploid level, and meiosis resulting in gametes of the next generation. The generations do not overlap. Let *x_ij_* be a diploid genotype made up of haplotypes *i* and *j*. Its frequency *p^s^* after selection (as an adult) can be calculated based on its frequency *p* before selection (as a zygote) and its absolute fitness *W*:

(1) $$p^{s}\left(x_{\mathrm{ij}}\right)=\frac{p\left(x_{\mathrm{ij}}\right)\cdot W\left(x_{\mathrm{ij}}\right)}{\sum_{\mathrm{ij}}p\left(x_{\mathrm{ij}}\right)\cdot W\left(x_{\mathrm{ij}}\right)}$$

Then, let *g_k_* be gamete of haplotype *k*. Its frequency *p* in the gamete pool can be calculated based on frequencies of adults and probabilities of recombination events:

(2) $$p\left(g_{k}\right)=\sum_{\mathrm{ij}}p^{s}\left(x_{\mathrm{ij}}\right)\cdot P_{ij\to k}^{r}$$

where $P_{ij\to k}^{r}$ is the probability of obtaining gamete g*_k_* from adult *x_ij_* in meiosis, with the given rates of recombination and crossover interference. Frequency *p* ^m^ of a given gamete after mutation can be calculated based on probabilities of mutation events:

(3) $$p^{m}\left(g_{k}\right)=\sum_{l}p\left(g_{l}\right)\cdot P_{l\to k}^{m}$$

where $P_{l\to k}^{m}$ is the probability of obtaining gamete *g_k_* from gamete *g_l_* via mutations. Finally, frequencies of zygotes in the next generation can be calculated based on frequencies of the corresponding gametes, given random mating:

(4) $$p_{t+1}\left(x_{\mathrm{ij}}\right)=p_{t}^{m}\left(g_{i}\right)\cdot p_{t}^{m}\left(g_{j}\right)$$

### 2.2 Genetic system and selection regime

Each genotype has either two (*A* and *B*) or three (*A*, *B*, and *C*) selected loci and a selectively neutral modifier locus (*M*) affecting RRs between the selected loci. The loci are arranged as *M*–*A*–*B*–*C*. Each selected locus is represented by two possible alleles: wild‐type (*A*, *B*, or *C*) and mutant (*a*, *b*, or *c*). The effect of the mutations on fitness is described by a standard multilocus model (Roze, 2009), as follows. The mutant alleles decrease fitness by *s* in the homozygous state and by *hs* in the heterozygous state (parameters *s* and *h* are referred to as “deleterious effect of mutation” and “dominance of mutation,” respectively). For simplicity, both *s* and *h* are equal for all selected loci. The interlocus interaction is multiplicative with epistasis (purely multiplicative selection is also considered as a specific case). When assumed, the epistasis is represented by three components: additive‐by‐additive (*e*_a×a_), additive‐by‐dominance (*e*_a×d_), and dominance‐by dominance (*e*_d×d_), all of which are modeled as multiplicative terms. Thus, the fitness of the genotype bearing *N*_he_ heterozygous mutations and *N*_ho_ homozygous mutations is (Roze, 2009):

(5) $$W={\left(1-hs\right)}^{N_{\text{he}}}\cdot {\left(1-s\right)}^{N_{\text{ho}}}\cdot {\left(1+e_{a\times a}\right)}^{P_{a\times a}}\cdot {\left(1+e_{a\times d}\right)}^{P_{a\times d}}\cdot {\left(1+e_{d\times d}\right)}^{P_{d\times d}}$$

Here, the powers *P*_a×a_, *P*_a×d,_ and *P*_d×d_ stand, respectively, for the number of additive‐by‐additive, additive‐by‐dominance, and dominance‐by‐dominance epistatic interactions. These numbers can be obtained through *N*_he_ and *N*_ho_, as follows (Roze, 2009):

(6) $$\begin{array}{c} P_{a\times a}=N_{\text{he}}\left(N_{\text{he}}-1\right)/2+2N_{\text{he}}N_{\text{ho}}+4\left[N_{\text{ho}}\left(N_{\text{ho}}-1\right)/2\right]\\ P_{a\times d}=N_{\text{he}}N_{\text{ho}}+4\left[N_{\text{ho}}\left(N_{\text{ho}}-1\right)/2\right]\\ P_{d\times d}=N_{\text{ho}}\left(N_{\text{ho}}-1\right)/2 \end{array}$$

### 2.3 Recombination strategies

Modifier alleles define RRs within the selected system (*r_S_*). For simplicity, in the three‐locus selected system, RRs between the adjacent selected loci are assumed to be equal (*r*_AB_ =* r*_BC_ = *r*_S_). The modifier locus is assumed to be either unlinked (*r*_MA_ = 0.5) or linked (*r*_MA_ = 0.05) to the selected system. The relations between modifier alleles are assumed to be purely codominant.

The modifier alleles confer various recombination strategies. We consider two types of strategies, implying that (a) all genotypes of the selected system have the *same* RR (constant strategies) and (b) different genotypes have *different* RRs, varying according to their fitness (FD‐strategies). Under FD‐strategy, RRs within the selected system (*r*_S_) negatively covary with genotype fitness (*W*). Specifically, the genotype with the highest fitness (*W*_max_) has the lowest recombination rate (*r*_min_) and vice versa. For genotypes with intermediate fitness values, RRs are obtained by linear interpolation:

(7) $$r_{S}\left(W\right)=\left\{\begin{array}{ccc} r_{\min}, & \text{for} & W=W_{\max}\\ r_{\min}+\left(r_{\max}-r_{\min}\right)\cdot \frac{W_{\max}-W}{W_{\max}-W_{\min}}, & \text{for} & W_{\min}<W<W_{\max}\\ r_{\max}, & \text{for} & W=W_{\min} \end{array}\right.$$

We assume that a precondition for the evolutionary advantage of FD recombination is variation in fitness among *recombination‐responsive genotypes* (double and triple heterozygotes). In this respect, when estimating the lowest and highest fitness values (*W*_min_ and *W*_max_), we took into account only such genotypes. However, in models with two selected loci, there exists only one recombination‐responsive genotype (double heterozygote), and such normalization would result in division by zero (Equation 8). In this case, we estimated the lowest (*W*_min_) and highest (*W*_max_) fitness values among *all genotypes*. In the majority of simulations, the magnitude of the plastic effect (∆ *= r*_max_ *–r*_min_) was put equal to 0.05; we also examined higher magnitudes, up to 0.5.

### 2.4 Criteria for comparison of recombination strategies

First and foremost, we compared alternative recombination strategies in terms of *individual selection*, based on the dynamics of modifier alleles (Kimura, 1956; Nei, 1967). Specifically, strategy *S*_1_ was regarded as evolutionarily more advantageous than strategy *S*_2_ if the modifier allele for *S*_1_ succeeded in the two following tests. First, it had to invade the population in which the modifier allele for *S*_2_ was nearly fixed (allele frequency of 0.95). Second, it had to resist, when it was nearly fixed itself, invasion by the modifier allele for *S*_2_. In both tests, the system was first allowed to reach the state of mutation–selection balance (which was diagnosed when allele frequencies at each selected locus changed by less than 10^–12^ per generation) with the monomorphic modifier locus, after which it evolved during 10,000 generations with the polymorphic modifier locus.

Aside from the dynamics of modifier alleles, we also compared recombination strategies in terms of the population mean fitness and population genetic variation. The population mean fitness was calculated as the fitness of all genotypes weighted by their frequencies. Population genetic variation (*v*) was calculated as the loci‐averaged standard deviation of allele frequencies within the selected system:

(8) $$v=\frac{1}{n}\sum_{i}\sqrt{p_{i}\left(1-p_{i}\right)}$$

where *p_i_* is allele frequency at the *i*‐th selected locus, and *n* is the number of selected loci.

### 2.5 Design of the numerical experiments

We examined selected systems with two and three selected loci; for each of them, two situations were considered: with unlinked (*r*_MA_ = 0.5) and linked (*r*_MA_ = 0.05) modifier, in order to address the potential effect of modifier linkage on the modifier‐allele dynamics (see for review: Korol et al., 1994; Otto, 2009). We scanned combinations of five selection parameters: deleterious effect of mutations (*s*), the dominance of mutations (*h*), and three epistatic components (*e*_a×a_, *e*_a×d_, and *e*_d×d_). For simplicity, both *s* and *h* were put equal for all selected loci. With respect to the deleterious effect, the mutations were scanned from almost neutral (*s* = 0.01, as a proxy for *s* ≈ 0) to lethal (*s* = 1), with a step of 0.1. With respect to the dominance, the mutations were scanned from purely recessive (*h = *0) to purely dominant (*h* = 1), with a step of 0.2. The epistatic components were initially scanned from –1 to 1, with a step of 0.1. Mutations were assumed to be unidirectional, with a rate of 10^–4^ per selected locus. No mutations were assumed at the modifier locus. In the first round of simulations, we assumed no crossover interference within the selected system (i.e., coefficient of coincidence *c* = 1). Then, in order to test for the effect of crossover interference on the obtained results, we additionally examined two other situations: with full positive interference, implying no double‐crossover events (*c* = 0), and with a considerable negative interference, implying an excess of double crossovers (*c* = 2). In these additional simulations, the additive‐by‐dominance epistasis was scanned with a step of 0.5, while the dominance‐by‐dominance epistasis was put equal to zero. The reason for treating the second (additive‐by‐dominance) and the third (dominance‐by‐dominance) epistatic components more roughly was that their effect appeared to be, respectively, one and two orders of magnitude weaker compared with that of the first (additive‐by‐additive) epistatic component.

For each system and each combination of selection parameters, we compared FD recombination with the optimal constant RR. The optimal constant RR within the selected system ($r_{S}^{*}$) was estimated as follows. First, the minimal RR (*r*_S_ = 0) was compared with one step higher RR (*r*_S_ = δ*r*). If modifier allele for the latter invaded, it was compared with that for one more step higher RR (*r*_S_ = 2δ*r*), and so on. These pair‐wise comparisons were conducted until the modifier allele for a higher RR failed to invade. Once this happened, the previous RR was regarded as a lower estimate for the optimal constant RR ($r_{S}^{*\text{low}}$). Second, we started with the maximal RR (*r_S_* = 0.5) and moved downward (*r_S_* = 0.5–δ*r*, *r_S_* = 0.5–2δ*r*, etc.) until modifier allele for a lower RR failed to invade. Upon this, the previous RR was regarded as a higher estimate for the optimal constant RR ($r_{S}^{*\text{high}}$). Then, we repeated the procedure between the obtained lower and higher estimates using the one order of magnitude smaller step, to obtain new, more accurate estimates. In total, we used three iterations, with steps equal to 0.01, 0.001, and 0.0001. The final lower and higher estimates differed by no more than 0.0001; the average between these two values was used as the optimal constant RR ($r_{S}^{*}$).

Once found, the optimal constant RR was compared with FD recombination. If zero RR was optimal, we compared it with FD‐strategy where RR varied from *r*_min_ = 0 to *r*_max_ = ∆*r*. If an intermediate RR was optimal, we compared it with three different FD‐strategies where RR varied: (a) *above* the optimal RR, from $r_{\min}=r_{S}^{*}$ to $r_{\max}=r_{S}^{*}+\Delta r$; (b) *below* the optimal RR, from $r_{\min}=r_{S}^{*}-\Delta r$ to $r_{\max}=r_{S}^{*}$; and (c) *around* the optimal RR, from $r_{\min}=r_{S}^{*}-\Delta r/2$ to $r_{\max}=r_{S}^{*}+\Delta r/2$. Hereafter, we refer to these three FD‐strategies as “recombination‐increasing” (denoted as “+FD”), “recombination‐decreasing” (“–FD”), and “fringe” (“±FD”), respectively.

The herein presented results are based on numerical simulations, which does not allow strict inferences about conditions that favor/disfavor FD recombination. Nevertheless, it is possible to discriminate parameter combinations leading to the alternative outcomes (favor vs. no favor) using numerical classification methods as a proxy, despite the fact that the considered model is purely deterministic and the described experimental design implies no stochasticity. Thus, to “quantify” the relative influence of the model parameters (the deleterious effect of mutations, their dominance, the epistatic components, the range of RRs under FD recombination, etc.) on the fate of FD recombination, we employed the tools of logit analysis.

## 3 RESULTS

### 3.1 Multiplicative selection

As expected, under *multiplicative* selection (*e*_a×a_ = *e*_a×d _= *e*_d×d_ = 0), two arbitrary constant RRs always remained neutral to one another in terms of the modifier‐allele dynamics (i.e., the modifier‐allele frequencies did not change regardless of their initial frequencies). Moreover, all constant RRs ensured the same equilibrium population mean fitness. Similarly, modifier alleles for FD recombination were neutral to those for the corresponding constant RR. This held for all examined systems: with two and three selected loci, and with the unlinked and linked modifier. Under *epistatic* selection, different constant RRs stopped being neutral one to another, which allowed estimating the optimal constant RR and comparisons with FD recombination.

### 3.2 Epistatic selection: FD recombination versus zero optimal constant RR

**Figure 1:**
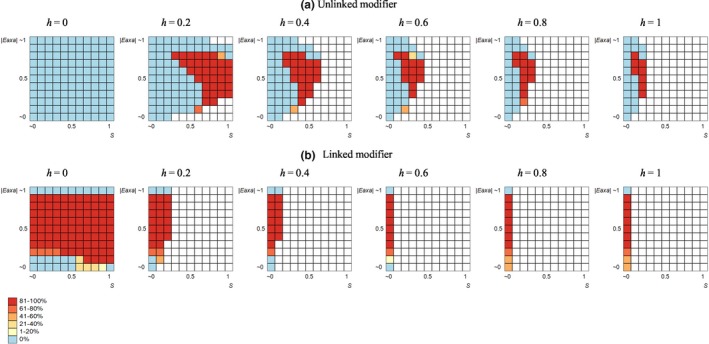
The evolutionary advantage of FD recombination over zero optimal constant RR: the effect of key selection parameters. The data stand for the system with three selected loci and no crossover interference within the selected system (though the pattern appeared to be strongly robust to crossover interference). In each heat map, *x* and *y* axes stand, respectively, for the deleterious effect of mutations (*s*) and the absolute value of negative additive‐by‐additive epistasis (|*e*_a×a_|). The colors stand for the proportion of cases where FD recombination is favored (as a ratio to the total number of cases with the given parameter combination). No color means that no cases with zero optimal constant RR were observed under the given parameter combination

As expected, selection for/against nonzero RRs was strongly affected by the sign of the epistasis. Zero optimal constant RR was observed in a vast area of the parameter space: always under positive and often (but not necessarily) under negative additive‐by‐additive epistasis. The proportion of cases with zero optimal constant RR tended to decrease with a higher dominance of deleterious mutations, in accordance with the results reported by Roze (2009). In the system with *two* selected loci, FD recombination was never favored over zero optimal constant RR; this holds for both systems with the unlinked and linked modifier locus. In contrast, in the system with *three* selected loci, FD recombination was nonrarely favored over zero optimal constant RR. A necessary (but not sufficient) condition for the evolutionary advantage of FD recombination was negative epistasis. Other influential parameters were the deleterious effect of mutations and their dominance; at that, considering the product *s⋅h* as a joint variable increased predictability of the outcomes, arguing for the crucial role of heterozygotes in the evolutionary advantage of FD recombination (Tables A1 and A2). With the *unlinked* modifier, FD recombination was favored under rather strong deleterious effects of mutations but intermediate negative additive‐by‐additive epistasis (so that the corresponding area of the parameter plane resembled a rightward‐curved sickle). Low dominance of deleterious mutations tended to mitigate selection for FD recombination (Figure 1a). With the *linked* modifier, zero optimal constant RR was observed in a smaller number of cases, typically under weak deleterious effects of mutations (unless the latter ones were purely recessive). Yet, almost in all such cases FD recombination was selected for, with the exception of those with weak negative additive‐by‐additive epistasis (Figure 1b).

In total, in the system with three selected loci, FD recombination was favored over zero optimal constant RR in ~23% and ~79% of cases with unlinked and linked modifiers, respectively. Perhaps, the higher proportion of cases where FD recombination was favored in the system with linked modifier originates from a longer association of the modifier allele with the selected haplotypes; this longer association mitigates the constraints imposed on epistasis—as it happens when a population practices less sex compared with panmixia (Otto, 2009). With higher magnitudes of the plastic effect, the above‐reported numbers increased, reaching ~30% and ~83% for the unlinked and linked modifier, respectively. At that, higher magnitudes tended to relax the above‐mentioned restriction on epistasis, making even strong negative additive‐by‐additive epistasis compatible with selection for FD recombination.

The above results were obtained for situations with no crossover interference within the selected system, which in principle should be considered as a special case rather than a rule. The results appeared to be strongly robust: The proportion of cases where FD recombination was favored under *c* = 0 and *c* = 2 deviated from that under *c* = 1 by <0.2%. Yet, crossover interference did affect quantitatively: Under lower values of *c*, the modifier allele for FD recombination invaded the population more easily.

Notably, although the overall number of cases where FD recombination was favored grew with the magnitude of the plastic effect, in rare cases, the large‐magnitude +FD‐strategy appeared less successful (when compared with zero optimal constant RR) than the small magnitude one. This gave rise to the natural question of whether an *optimal* magnitude of the plastic effect exists. To address it, we compared +FD‐strategies with different magnitudes and indeed found that in such cases a modifier allele for a certain intermediate magnitude displaced those for both smaller and larger magnitudes.

We also compared FD recombination and zero optimal constant RR in terms of the population mean fitness and the population genetic variation. Under negative epistasis, a population with FD recombination had a higher mean fitness and a lower population genetic variation than the same population with zero optimal constant RR. These differences were very small but held regardless of whether FD recombination was favored or disfavored. Yet, in the cases where FD recombination was favored, the difference was much less pronounced (~10^–9^–10^–13^ for the population mean fitness and ~10^–7^–10^–12^ for the population genetic variation) compared with those where FD recombination was disfavored (~10^–7^–10^–10^ and ~10^–3^–10^–6^, respectively). The difference was higher with the large magnitude of the plastic effect (by 1–2 orders, compared with the small magnitude). Apparently, whenever FD recombination was favored, it shifted upward the population mean RR. Yet, this shift was very small: up to ~10^–5^–10^–3^ in the model with the unlined modifier, and up to ~10^–4^–10^–2^ in the model with the linked modifier. The reason is that the lower‐fitness genotypes (i.e., those displaying higher RRs under FD recombination) remained very rare in the population subjected to purifying selection. As a consequence, selection for FD recombination was also very weak: The invasion of the modifier allele for FD recombination could be stopped by burdening this allele with a multiplicative fitness‐decreasing effect of ~10^–8^–10^–10^ in the model with linked modifier and ~10^–10^–10^–12^ in the model with the unlinked modifier.

### 3.3 Epistatic selection: FD recombination versus intermediate optimal constant RR

**Figure 2:**
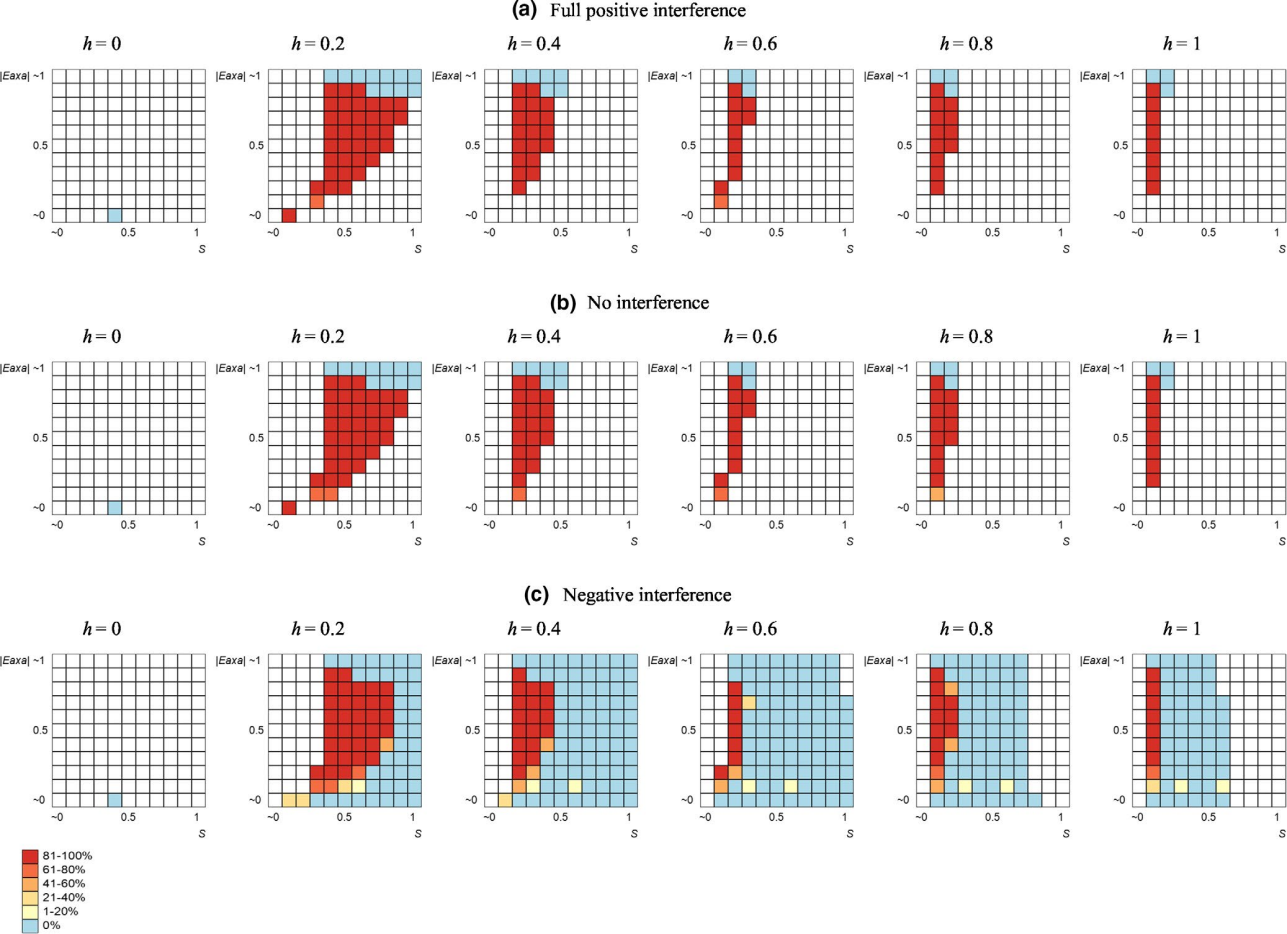
The evolutionary advantage of FD recombination over intermediate optimal constant RR: the effect of key selection parameters and crossover interference within the selected system. The data stand for the system with three selected loci and linked modifier, +FD‐strategy. In each heat map, *x* and *y* axes stand, respectively, for the deleterious effect of mutations (*s*) and the absolute value of negative additive‐by‐additive epistasis (|*e*_a×a_|). The colors stand for the proportion of cases where FD recombination is favored (as a ratio to the total number of cases with the given parameter combination). No color means that no cases with intermediate optimal constant RR were observed under the given parameter combination

Intermediate optimal constant RRs were found only under negative epistasis, as predicted by the theory (Barton, 1995; Charlesworth, 1990; Feldman et al., 1980; Gabriel et al., 1993; Kondrashov, 1984; Lynch et al., 1995; Otto & Barton, 1997; Otto & Feldman, 1997). Whenever the optimal constant RR was intermediate, it was compared with three FD‐strategies: with recombination‐increasing, recombination‐decreasing, and “fringe” ones (i.e., with RRs varying above, below, and around the optimal constant RR, respectively). In the system with *two selected loci*, FD recombination was never favored. In contrast, in the system with *three selected loci*, the +FD‐strategy appeared to be favored in a predominant proportion of cases (~76%), with the exception of marginal (either too low or too high) values of selection intensity and additive‐by‐additive epistasis. The evolutionary advantage of the ±FD‐strategy was sporadic (~1%), and totally absent for –FD‐strategy. Again, the above‐presented results stand for the situation with no crossover interference (*c* = 1). Our simulations showed strong robustness of the revealed evolutionary advantage of FD recombination to crossover interference, either positive (c = 0) or negative (*c* = 2), similar to the situations with zero optimal constant RR. Noteworthy, negative interference considerably expanded the parameter area with intermediate optimal constant RR (Figure 2).

### 3.4 FD‐induced modulation of modifier linkage to the selected loci

**Figure 3:**
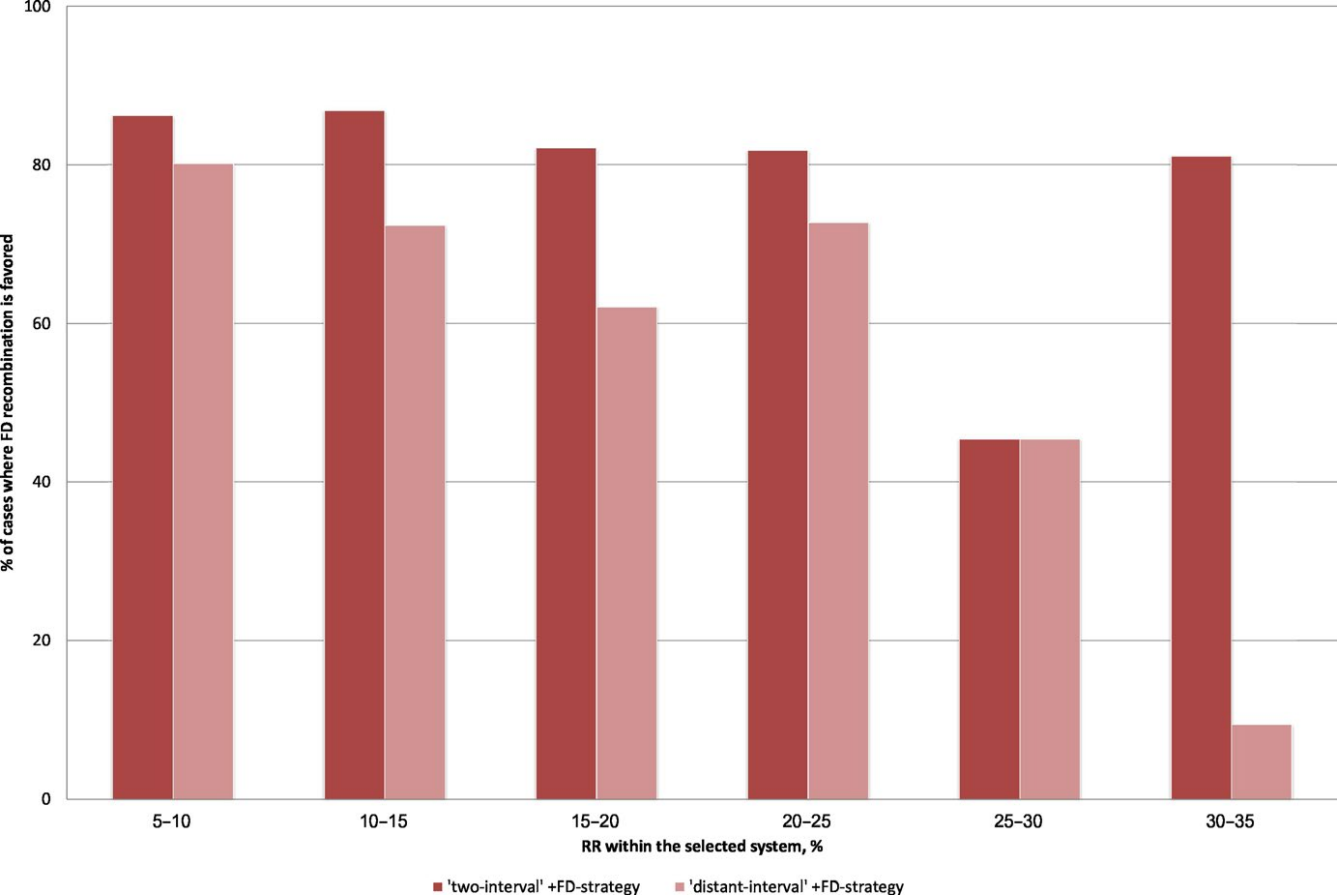
The effect of FD‐induced modulation of modifier linkage on the evolutionary advantage of FD recombination over intermediate optimal constant RR. The data stand for the system with three selected loci and linked modifier, +FD‐strategy. The “distant‐interval” +FD‐strategy can still be favored, although it is less advantageous than the “two‐interval” +FD‐strategy. This suggests that FD‐induced modulation of modifier linkage may play a certain role in systems with linked modifier, decreasing with linkage intensity between the selected loci

When testing for the evolutionary advantage of FD recombination in the system *M*–*A*–*B*–*C*, we assumed the effect of genotype fitness on RR between the selected loci, that is, on *r*_AB_ and *r*_BC_, but not on *r*_MA_. Nevertheless, despite this assumption, FD recombination between the selected loci (*r*_AB_) gives rise to variation in RR between the modifier locus and the second selected locus (*r*_MB_). Indeed, *r*_MB_ depends on both *r*_MA_ and *r*_AB_; thus, the variation in *r*_AB_ under FD recombination inevitably leads to some variation in *r*_MB_, even though *r*_MA_ is constant. This variation of the modifier linkage to a part of the selected loci can affect the evolutionary advantage of FD recombination over the optimal constant RR because the value of the optimal RR by itself depends on the distance between the modifier and the selected (Korol et al., 1994; Otto, 2009). To address this issue, we additionally examined an FD‐strategy implying the effect of genotype fitness only on *r*_BC_ but not *r*_AB_ (hereafter, it is referred to as “distant‐interval” FD recombination). For this strategy, we assumed twice higher magnitude of the plastic effect, in order to ensure a “more honest” comparability with the earlier considered “two‐interval” strategy. Such “distant‐interval” +FD‐strategy was favored over the intermediate optimal constant RR in a smaller proportion of cases (~51%) than the “two‐intervals” +FD‐strategy (~76%). At that, the difference between these two strategies tended to grow with the optimal constant RR in the distant interval (Figure 3).

## 4 DISCUSSION

### 4.1 Abandon‐ship or differential disruption of low‐ versus high‐fitness genotypes?

Empirical studies have demonstrated that RRs can be sensitive to genotype fitness. However, despite the progress in the theoretical explanation of the evolution of FD recombination in haploids (Agrawal et al., 2005; Gessler & Xu, 2000; Hadany & Beker 2003a,2003b; Wexler & Rokhlenko, 2007), the so far suggested models encountered difficulties in extending the explanations to diploids. We find it reasonable to distinguish between two forms of FD recombination, according to the “target” genome region. The first one implies that genotype fitness affects only RR *between the modifier locus and the selected system*. The evolution of such a “selfish” FD recombination is relatively easy to explain. If a modifier allele is capable of “evaluating” its current genetic environment and tends to recombine from the low‐fitness chromosome, it will increase its chances of surviving and spreading, a mechanism referred to as “abandon‐ship” (Agrawal et al., 2005; Hadany & Beker, 2003b). This mechanism seems to be inefficient in diploids (Agrawal et al., 2005; Rybnikov et al., 2017). In the current study, we considered another form of FD recombination, implying that genotype fitness affects only RRs *within the selected system* (we call it “altruistic” FD recombination, in opposition to “selfish” FD recombination). We examined “selfish” FD recombination only in an additional experiment, aimed to test for the consistency of our models with those focusing on the “abandon‐ship” mechanism. In this additional experiment, the modifier allele for “selfish” FD recombination was always neutral in relation to the optimal constant RR allele, which is consistent with the analytical solution of Agrawal et al. (2005). Moreover, any *arbitrary* modifier alleles were always neutral one to another if they conferred the same RRs within the selected system, regardless of their effect on RR between the modifier locus and the selected system. The key result of our main simulations, conducted with “altruistic” FD recombination, showed that this strategy can be favored in diploids, which extends to diploids the results previously obtained Hadany and Beker (2003b) in their haploid models with the unlinked modifier. With this strategy, FD recombination tends to disrupt lower‐fitness selected genotypes more intensively than higher‐fitness ones thereby reducing the inherent costs of recombination. Recent theoretical analysis shows that analogous fitness‐dependent strategies can be evolutionary advantageous also in relation to other sources of genetic variation (Ram, Altenberg, Liberman, & Feldman, 2018).

### 4.2 FD recombination as a trade‐off

We observed selection for “altruistic” FD recombination in models with three but not two selected loci. The reason is that the plasticity of RRs within the selected system implies not only benefits (differential rate of disruption of lower‐ vs. higher‐fitness genotypes) but also some costs. Indeed, the population mean RR established under FD recombination may depart from the optimal constant RR. In such a situation, the fate of the modifier allele for FD recombination is determined by a trade‐off between the benefit of RR plasticity and the cost of such departure. While the cost emerges in any system, with either two or three selected loci, the benefit can arise only in systems with at least three selected loci; otherwise, no variation in fitness would be possible among double heterozygotes. With respect to the mentioned trade‐off, we suggest that whenever FD recombination was favored in our simulations, the benefit of RR plasticity outbalanced its cost.

It turned out that with the growing magnitude of the plastic effect, the benefit usually grew “faster” than the cost, as reflected by the proportion of cases where FD recombination was favored. In some cases, we observed an intermediate optimal magnitude, which suggests that the cost outbalanced starting from a certain threshold magnitude and further argues that the evolutionary advantage of FD recombination is a kind of trade‐off. One more argument is the intriguing discrepancy between +FD‐strategy and –FD‐strategy in terms of their evolutionary advantage over the intermediate optimal constant RR: ~76% and <1%, respectively. This can be explained by the fact that under purifying selection, the major part of the population is represented by the wild‐type genotypes (in our simulations, assuming a relatively high mutation rate of 10^–4^ per locus, the mutant allele frequencies never exceeded 0.2%). Thus, the –FD‐strategy (which decreases RR in higher‐fitness genotypes) strongly moves the population mean RR downwards making –FD‐strategy unfavorable.

Although we consider the differential rate of disruption of lower‐ versus higher‐fitness genotypes as the key mechanism driving the evolution of FD recombination in diploids, it may give rise to FD‐induced modulation of the modifier linkage to the selected system, also contributing to evolutionary advantage of FD recombination. Such modulation occurs under certain conditions: (a) The selected system consists of at least three linked loci, (b) the modifier locus is linked to the selected system, and (c) at least three of the selected loci are located from one side of the modifier. The results obtained for unlinked modifier are free of these complications.

### 4.3 Fitness dependence as an “evolutionary rescue” for recombination

The evolutionary advantage of FD recombination in diploids was observed in several population genetics models: mutation‐selection balance (the herein presented results), cyclical selection (Rybnikov et al., 2017), and Red Queen dynamics (Rybnikov, Frenkel, Fahima, & Korol, 2018), which argues for the universality of the underlying mechanism. Remarkably, in all three mentioned population genetics models, FD recombination was shown to be favored under certain parameter combinations even if *any constant* nonzero RR was disfavored. This indicates that assuming FD recombination enables selection for nonzero RRs under much milder constraints on the key model parameters (e.g., population size, selection intensity, epistasis, etc.) compared with those in classical models with constant RRs (Barton, 1995; Otto & Barton, 1997, 2001; Otto & Feldman, 1997). Thus, the “recombination‐supporting potential” of FD recombination, first demonstrated by Gessler and Xu (2000) for haploids, can be extended also to diploids. Notably, the same pattern was revealed for the rate of sex, which is also long known to exhibit fitness dependence (see for review: Ram & Hadany, 2016). Specifically, FD sex was shown to be favored over asexual reproduction even if *any constant* nonzero rate of sex was disfavored (Mostowy & Engelstädter, 2012).

While the “abandon‐ship” mechanism of FD recombination was shown to be efficient only in haploids, the differential rate of disruption of lower‐ versus higher‐fitness genotypes can be evolutionarily advantageous in both haploids and diploids. We speculate that FD recombination could first appear in haploids as an “invention” by some “selfish” recombination‐controlling alleles, which spread by exploiting the “abandon‐ship” benefits (Gessler & Xu, 2000; Otto, 2009). Later on, such alleles probably expanded the ability to affect RR in an FD manner to other genome regions. Once this happened, FD recombination stopped being entirely dependent on the “abandon‐ship” benefits, and could also evolve in diploids, where the “abandon‐ship” mechanism does not work alone. Such extension can be considered as a transformation of “effect” into “function” (Maynard Smith, 1982) in the course of evolution of recombination, fitting well the relay‐race principle (Ratner, 1990).

### 4.4 Concluding remarks

Apparently, the examined systems with two or three selected loci having equal effects on fitness are too simplified to be realistic. Yet, our rationale was to start with the simplest possible model, in order to test the principle. More complex systems (with a larger number of selected loci and/or with loci differing in their effect on fitness) are not only more realistic but also seem more promising from the viewpoint of selection for recombination in general and for FD recombination specifically, due to higher variation in fitness in such systems. Presumably, important insights will also be brought into understanding the evolution of FD recombination if the latter is allowed to coevolve together with the architecture of the selected system, similar to what was shown in models with constant reproduction strategies (Lohaus, Burch, & Azevedo, 2010; Whitlock, Peck, Azevedo, & Burch, 2016).

## CONFLICT OF INTEREST

None declared.

## AUTHOR CONTRIBUTIONS

Sviatoslav Rybnikov participated in designing the numerical experiments, developed the algorithms, wrote the codes for the simulations, analyzed the results, and drafted the manuscript. Zeev Frenkel participated in designing the numerical experiments and developing the algorithms. Abraham B. Korol introduced the idea of the study, designed the numerical experiments, participated in drafting the manuscript, and coordinated the study.

## Data Availability

The codes used for the simulations, as well as the corresponding input and output files, are available at Dryad, https://doi.org/10.5061/dryad.kh189322c.

## APPENDIX 1

**Table A1:** The relative influence of model parameters on the evolutionary advantage of FD recombination over zero optimal constant RR: the system with three selected loci and unlinked modifier

Variable	Model 1		Model 2	
	*B*	Wald	*B*	Wald
Deleterious effect of mutations (*s*)	5.36	2,536.66	35.32^a^	4,269.04^a^
Dominance of mutations (*h*)	5.34	2,739.63		
Additive‐by‐additive epistasis (*e*_a×a_)	−1.22	268.01	−1.12	128.23
Additive‐by‐dominance epistasis (*e*_a×d_)	−0.02	0.88	−0.06	2.42
Crossover interference (*c*)	−0.05	4.15	−0.09	7.44
Magnitude of the plastic effect (Δ*r*)	0.92	70.79	1.63	121.92
Constant	−6.00	3,148.97	−5.17	2,419.19
Total number of cases	17,205			
Correctly predicted cases, %	76.5		88.5	

a The product *s⋅h* is used as a joint variable.

**Table A2:** The relative influence of model parameters on the evolutionary advantage of FD recombination over zero optimal constant RR: the system with three selected loci and linked modifier

Variable	Model 1		Model 2	
	*B*	Wald	*B*	Wald
Deleterious effect of mutations (*s*)	0.85	67.49	10.21^a^	13.71^a^
Dominance of mutations (*h*)	0.55	24.94		
Additive‐by‐additive epistasis (*e*_a×a_)	0.19	4.63	0.27	8.95
Additive‐by‐dominance epistasis (*e*_a×d_)	−0.10	7.30	−0.10	6.80
Crossover interference (*c*)	−0.002	0.00	−0.002	0.004
Magnitude of the plastic effect (Δ*r*)	0.63	18.12	0.64	18.01
Constant	1.03	135.29	1.39	338.96
Total number of cases	8,811			
Correctly predicted cases, %	81.2		81.2	

a The product *s⋅h* is used as a joint variable.
